# Do mitochondria donate membrane to form autophagosomes or undergo remodeling to form mitochondrial spheroids?

**DOI:** 10.1186/2045-3701-4-65

**Published:** 2014-11-14

**Authors:** Wen-Xing Ding, Eeva-Liisa Eskelinen

**Affiliations:** Department of Pharmacology, Toxicology and Therapeutics, University of Kansas Medical Center, MS 1018, 3901 Rainbow Blvd, Kansas City, KS 66160 USA; Department of Biosciences, Division of Biochemistry and Biotechnology, The University of Helsinki, 00014 Helsinki, Finland

## To the Editor

We read with great interest the recent paper by Cook et al. [1] in which the authors described a striking mitochondrial vesicle structure induced by some anti-cancer drugs (including Fulvestrant, an antiestrogen drug, or Imatinib, a tyrosine kinase inhibitor) in cultured cancer cells. Using electron microscopy immuno-gold labeling, they further found that some of these mitochondrial vesicles seemed to be labeled with microtubule-associated protein 1 light chain 3 (LC3), a marker for autophagosomes/autolysosomes. Therefore the authors concluded that mitochondria directly donate their membrane to form autophagosomes. We recently reported a similar mitochondrial structure change, which we named mitochondrial spheroids [2, 3]. Mitochondrial spheroids are unique mitochondrial structures, which have a ring or cup-like morphology. They look similar to autophagosomes with the interior, or lumen, being surrounded by four mitochondrial membranes and squeezed mitochondrial matrix [3]. Intriguingly, we also demonstrated by confocal microscopy and immuno-gold EM analysis that mitochondrial spheroids are acidic compartments that have acquired lysosomal markers. Mitochondrial spheroids are found in cultured immortalized cells and cancer cells when they are subjected to CCCP-induced mitochondrial depolarization, a widely used mitophagy model in vitro. More importantly, we also found an increase in the number of mitochondrial spheroids in acetaminophen-treated mouse liver. Mechanistically, we found that the formation of mitochondrial spheroids requires mitochondrial fusion proteins mitofusin 1 or mitofusin 2 and is negatively regulated by Parkin, an ubiquitin E3 ligase that induces mitophagy in vitro [4]. The altered mitochondrial structures in the study by Cook et al. are very similar to mitochondrial spheroids, which seems to suggest that formation of mitochondrial-formed autophagosomes/mitochondrial spheroids could be a common mitochondrial adaptation response to various mitochondrial stresses. However, there are fundamental differences in these two studies in terms of data interpretation and conclusions. One key question is whether mitochondria directly donate their membrane to form autophagosomes. The evidence for the notion that mitochondria donate their membranes to form autophagosomes was based on immuno-gold labeling using an LC3 antibody, which Cook et al. used to show that LC3 positive membranes were closely associated with mitochondrial vesicles. While these data could suggest that mitochondrial membranes were utilized to form autophagosomes, the closely-associated LC3 positive membrane could also be due to the fusion of mitochondrial vesicles with an autophagosome/autolysosome. Alternatively, it is also possible that LC3 positive membrane next to a mitochondrial vesicle could reflect the process where a mitochondron is enveloped by an autophgosome. However, it should be noted that there was no difference in the extent of formation of mitochondrial spheroids in wild type and Atg5- or Atg7-deficient cells, suggesting that the formation of mitochondrial spheroids is independent of canonical autophagy machinery [2]. Cook et al. did not test the dependency on Atg7, of the mitochondrial-formed autophagosome structures in the drug-treated cancer cells. Our second concern is the notion that mitochondrial-formed autophagosomes represent a novel mechanism of Parkin-associated mitophagy. Cook et al. found that there were very high levels of Pink1 in the cytosolic fractions in LCC9 breast cancer cells with barely detectable levels on the mitochondria after drug treatment. This is quite intriguing since it is generally thought that Pink1 is a mitochondrial serine/threonine protein kinase [5–7]. Moreover, it is well known that Parkin has a tumor suppressor function and is generally silenced and its expression is lost in many immortalized and transformed cancer cells such as HeLa cells [8–10]. Many of the Parkin-mediated mitophagy studies in cultured cells rely on the ectopical overexpression of Parkin [9, 11]. Therefore, it is very interesting that Cook et al. found that LCC9 breast cancer cells had endogenous Parkin expression although it was unclear how these LCC9 cancer cells were different from other immortalized/cancerous cells. Moreover, it was also not clear how many of the mitochondrial-formed autophagosomes were labeled Parkin positive since no quantitative data were provided. Another unresolved issue for both mitochondrial spheroids and mitochondrial-formed autophagosomes are their unclear physiological roles. While we have proposed that the formation of mitochondrial spheroids may represent an alternative mechanism for regulating mitochondrial homeostasis and thus to limit drug-induced cell death, more future studies are definitely needed to further clarify this notion by using genetic mitofusin 1 or mitofusin 2 knockout cells in which mitochondrial spheroids cannot form. In the study by Cook at al, it was not clear whether the formation of the mitochondrial-formed autophagosomes served as mitochondrial quality control to protect the cells from anti-cancer drug-induced cell death since this aspect was not addressed. Taken together, it seems that more studies are needed to further confirm whether mitochondria donate their membrane to form autophagosomes as proposed in the Cook et al. study, or whether mitochondria are turned over by forming spheroids as proposed by our study [2, 3], or whether both alternatives are possible depending on the context. However, it can be concluded that certain drugs that may damage mitochondria can induce dramatic mitochondrial structural remodeling to form vesicular structures/mitochondrial spheroids. However, the physiological or pathological significance of these unique mitochondrial structures remains to be further studied.
